# Urokinase Salvage Therapy for Recurrent Chronic Subdural Hematoma Caused by Dual Antiplatelet Therapy

**DOI:** 10.7759/cureus.45320

**Published:** 2023-09-15

**Authors:** Jing Bao, Rui Sun, Zhenjiang Pan, Shepeng Wei

**Affiliations:** 1 Neurosurgery, Shidong Hospital of Yangpu District, Shanghai, CHN

**Keywords:** urokinase, subdural injection, salvage therapy, dual antiplatelet therapy, chronic subdural hematoma, catheter obstruction

## Abstract

Chronic subdural hematoma (cSDH) is one of the most common neurosurgical conditions among older adults. Here, we describe a case of recurrent cSDH resulting from seven days of dual antiplatelet therapy. A 76-year-old woman reported a headache resembling a chronic tension-type headache for the last 10 days. MRI revealed a right parietal cSDH and left frontoparietal cSDH. Single burr-hole aspiration and irrigation technique with continuous closed subdural drainage was performed bilaterally under general anesthesia. The patient experienced two bouts of transient ischemic attack and received seven days of dual antiplatelet therapy. On the 12th day after the initial surgery, the patient underwent another operation to re-evacuate the cSDH. Considering that the color of the output fluid persisted as oil-black and the average net output was 12.5 cc/day, we decided to use urokinase to restore the patency of the drainage catheter. In the early postoperative phase after the second surgery, a total of 20,000 units of urokinase was injected into the subdural space. On the 10th postoperative day, the patient was discharged home. In patients with cSDH presenting with obvious postoperative hematoma residue, the routine use of subdural injection of urokinase could be a new direction in cSDH management.

## Introduction

Chronic subdural hematoma (cSDH) is one of the most common neurosurgical conditions among older adults. Surgical treatment is the main method for symptomatic patients, with single burr-hole surgery being the most fundamental neurosurgical procedure.

The previous surgical treatment method for cSDHs at our hospital was single burr-hole irrigation with continuous closed subdural drainage. However, a modified burr-hole drainage method called single burr-hole aspiration and irrigation with continuous closed subdural drainage (SBAID) was introduced at our hospital in 2016 and has been used as a standard operation for cSDH for the last six years [1]. It is a variation of burr-hole drainage with irrigation that adds aspiration during the irrigation process and allows large debris from the cSDH content to be suctioned out. During the procedure, we chose Wei’s point as the burr hole and not the typical parietal eminence [1]. Although the recurrence of cSDH was 5-30% in surgically treated patients [2-4], our results showed a recurrence rate of only 2% in patients treated with SBAID as SBAID is the simplest neurosurgical operation. Furthermore, the recurrence of cSDH can have a large negative impact on surgeons, while re-operation may be more stressful for patients.

Although urokinase therapy for outcomes in patients with cSDH has been reported, we have no experience using urokinase for cSDH [5]. Here, we describe a case of recurrent cSDH caused by seven days of dual antiplatelet therapy that was cured by combining re-operation with a subdural injection of urokinase.

## Case presentation

**Figure 1:**
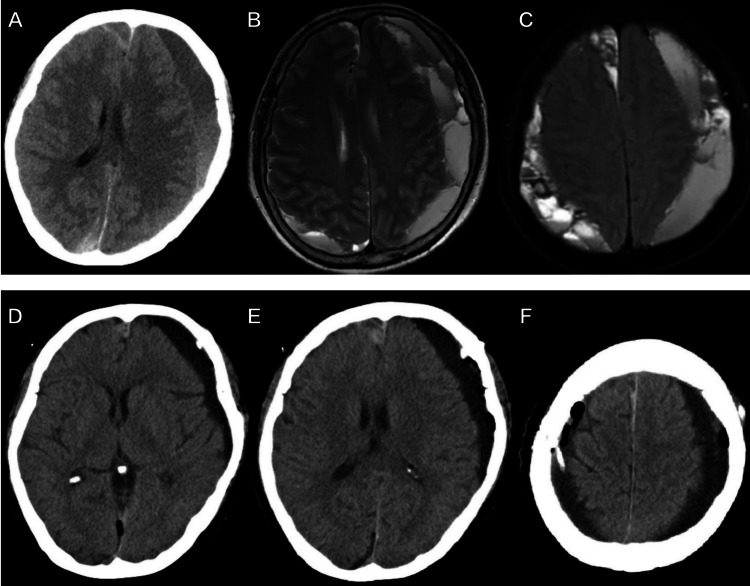
Preoperative CT and MRI axial brain images. Upper row (A, B, C): Preoperative neuroimaging showing bilateral chronic subdural hematoma, wherein the right hematoma is confined to the parietal lobe and the left hematoma is a typical hematoma covering the frontoparietal area, with a midline shift of 8.7 mm. Lower row (D, E, F): Postoperative CT (day two) demonstrating a left low-density shadow with a midline shift of 4.3 mm.

A 76-year-old woman presented with bilateral cSDH. She reported a headache resembling a chronic tension-type headache over the last 10 days. MRI revealed a right parietal cSDH and a left frontoparietal cSDH (Figure 1). SBAID was performed bilaterally under general anesthesia, as described previously. The left entry point was Wei’s point, which is located approximately 0.5 cm medial to the superior temporal line and 1 cm anterior to the coronal suture [1]. The whole procedure was uneventful, and the estimated blood loss was only 20 mL.

**Figure 2:**
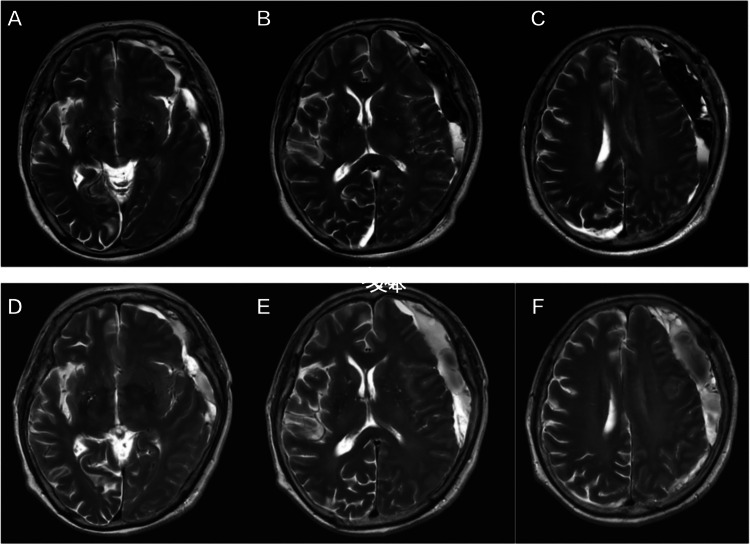
Axial MRI images after the first operation. Upper row (A, B, C): Postoperative MRI (day seven) indicating a massive recurrent left chronic subdural hematoma (cSDH) with midline shift. Lower row (D, E, F): Postoperative MRI (day 14) revealing the recurrent left cSDH with no improvement in the midline shift.

After surgery, the patient reported being fully recovered up to the fourth day (Figure 1). On the fifth day, she developed a sudden onset of Broca’s aphasia without hemiparesis, followed by normal neurological status one hour later. To prevent another stroke, the patient underwent dual antiplatelet therapy with acetylsalicylic acid (100 mg/day) and clopidogrel (75 mg/day). An MRI performed on the seventh day after surgery excluded acute cerebral infarction and showed nearly complete resolution of the right cSDH and persisting subdural fluid with a significant mass effect (Figure 2). Dual antiplatelet therapy was stopped to avoid expansion of the hematoma, and the patient was discharged. Four days later, she again presented with aphasia, and dual antiplatelet therapy was restarted. Five days later, another MRI showed significant left-side cSDH (Figure 2). Dual antiplatelet therapy was again stopped to avoid continued expansion of the hematoma.

**Figure 3:**
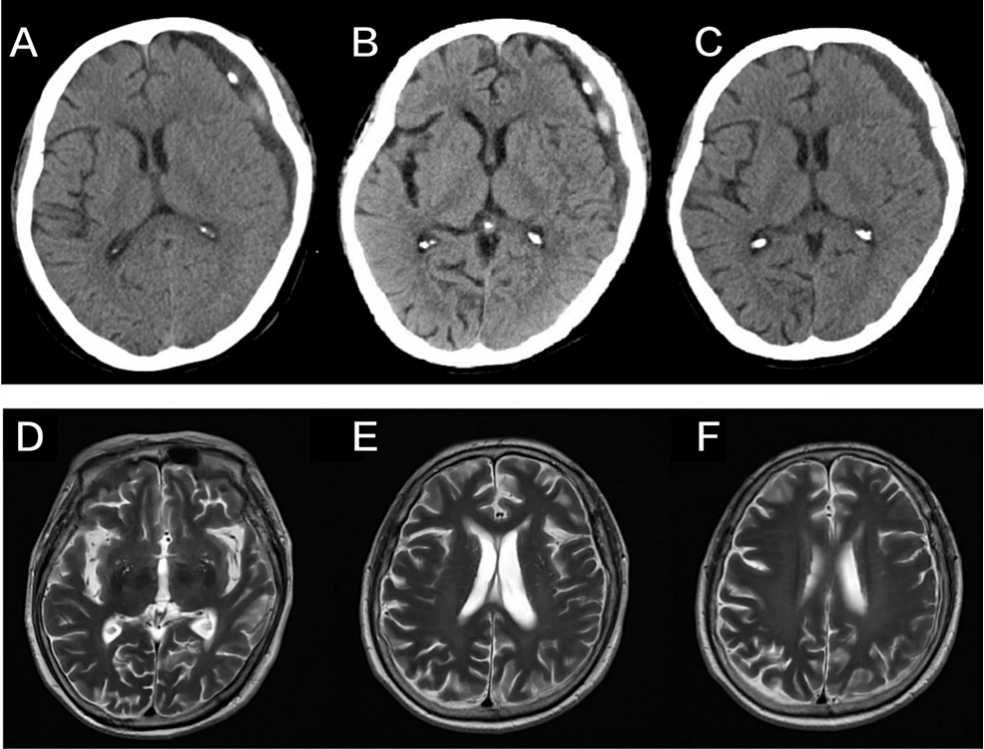
Axial brain images after the second operation. Upper row (A, B, C): Postoperative CT showing the changes in the chronic subdural hematoma. A: On day two, residual hematoma and a high-density shadow were detected. B: On day four, the residual hematoma and high-density shadow persisted even after the administration of 20,000 units of urokinase. C: On day 13, no significant mass effect or high-density shadow was observed. Lower row (D, E, F): Postoperative MRI (day 113) demonstrating normal brain structure without evidence of any subdural fluid collection.

On the 12th day after the first surgery, the patient underwent another operation to re-evacuate the left cSDH, and the left entry point was still Wei’s point. During surgery, the oil-black liquid of the cSDH was completely evacuated. Two days after the second surgery, she was found to be neurologically intact, and postoperative CT revealed decreased subdural space with mixed density (Figure 3A). However, the color of the output fluid was still oil-black and the average net output was only 12.5 cc/day; therefore, we decided to use urokinase therapy to restore the patency of the drainage catheter. Saline (5 mL) containing 10,000 units (U) of urokinase (Techpool, Guangzhou, China) was injected slowly into the hematoma cavity through the catheter, and another 3 mL of saline was used to flush the catheter. On the second day, another 10,000 U of urokinase was injected, as described above. The average net output increased to 32.6 cc/day during the next two days after the use of urokinase (Figure 3B). On the fourth postoperative day, the catheter was removed. Finally, the patient was discharged on the 10th postoperative day (Figure 3C). During the next 13 weeks, the patient did not receive any antiplatelet therapy, and no recurrent stroke or transient ischemic attack (TIA) was reported. Follow-up MRI on the 113th postoperative day demonstrated complete resolution of bilateral cSDH, and the patient was neurologically intact (Figures 3D-3F).

## Discussion

SBAID is the simplest neurosurgical operation. The cSDH recurrence rate after using SBAID was 2% in our hospital [1]. A study by Ou et al. that assessed the use of an exhaustive drainage strategy reported a recurrence rate of only 1.9%, which is the lowest for a large cohort [5].

Patients with symptomatic cSDH may present with headache, hemiplegia, cognitive decline, and eventually drowsiness or coma [6]. cSDHs rarely present with symptoms mimicking TIAs [7]. Our patient experienced two episodes of TIA and received seven days of dual antiplatelet therapy. An acute ischemic stroke was ruled out after two diffusion-weighted imaging scans.

Recurrence of cSDH after surgical evacuation is a serious problem that is encountered by neurosurgeons in 5-30% of cases [2-4]. In our patient, the bilateral cSDH, large volume of the postoperative hematoma cavity, and use of dual antiplatelet therapy may have all contributed to the recurrence of cSDH. Additionally, the impact of cSDH recurrence on a surgeon is no less than the impact of re-operation on a patient.

Urokinase administration in the subdural space was first reported by Arginteanu et al. in 1999 [8]. In that study, a 48-year-old woman who presented with recurrent cSDH was treated with urokinase administration via a subdural drain, which led to the resolution of the patient’s cSDH. The exhaustive drainage strategy in the study by Ou et al. included the use of urokinase in 53.6% of their patients [5]. The study showed that urokinase was safe and effective for improving drainage and greatly reducing recurrence. Although there were reports of the successful treatment of cSDH using urokinase, we had no experience with using urokinase in the subdural space. Therefore, we employed this method for the first time with a growth mindset.

We injected urokinase twice into the subdural space, and no hemorrhage complications occurred. Thus, the use of subdural injections of urokinase seems to be safe and a new direction in cSDH management. Lastly, it should be noted that the volume of the postoperative hematoma cavity was only 50 mL after the second SBAID, and the patient exhibited no symptoms. The residual hematoma might have spontaneously resolved several weeks later. Additionally, the dose of 20,000 U of urokinase was too small to cause intracranial hemorrhage.

## Conclusions

In patients with cSDH presenting with obvious postoperative hematoma residue, the routine use of subdural injections of urokinase could be a new direction in cSDH management. The existing evidence for the use of urokinase in cSDH is preliminary and not verified. Therefore, prospective, multicenter, randomized controlled studies are warranted to confirm the benefit and safety of urokinase as well as to identify the optimal agent and dosing regimen.
